# Crotamine induces browning of adipose tissue and increases energy expenditure in mice

**DOI:** 10.1038/s41598-018-22988-1

**Published:** 2018-03-22

**Authors:** Marcelo P. Marinovic, Joana D. Campeiro, Sunamita C. Lima, Andrea L. Rocha, Marcela B. Nering, Eduardo B. Oliveira, Marcelo A. Mori, Mirian A. F. Hayashi

**Affiliations:** 1 Departamento de Farmacologia, Escola Paulista de Medicina (EPM), Universidade Federal de São Paulo (UNIFESP), São Paulo, SP Brazil; 2 Departamento de Biofísica, Universidade Federal de São Paulo (UNIFESP/EPM), São Paulo, SP Brazil; 3 Departamento de Bioquímica e Imunologia, Universidade de São Paulo (USP-RP), Ribeirão Preto, Brazil; 4 Departamento de Bioquímica e Biologia Tecidual, Universidade Estadual de Campinas (UNICAMP), Campinas, SP Brazil

## Abstract

Crotamine, originally isolated from rattlesnake venom, has been extensively studied due to its pleiotropic biological properties, and special attention has been paid to its antitumor activity. However, long-term treatment with crotamine was accompanied by a reduction in animal body weight gain and by increases in glucose tolerance. As cancer is commonly associated with cachexia, to preclude the possible cancer cachexia-like effect of crotamine, herein this polypeptide was administered in healthy wild-type C57/BL6 mice by the oral route daily, for 21 days. Reduced body weight gain, in addition to decreased white adipose tissue (WAT) and increased brown adipose tissue (BAT) mass were observed in healthy animals in the absence of tumor. In addition, we observed improved glucose tolerance and increased insulin sensitivity, accompanied by a reduction of plasma lipid levels and decreased levels of biomarkers of liver damage and kidney disfunctions. Importantly, long-term treatment with crotamine increased the basal metabolic rate *in vivo*, which was consistent with the increased expression of thermogenic markers in BAT and WAT. Interestingly, cultured brown adipocyte cells induced to differentiation in the presence of crotamine also showed increases in some of these markers and in lipid droplets number and size, indicating increased brown adipocyte maturation.

## Introduction

Metabolism is regulated by a balance between energy expenditure and food intake^1^. In mammals, this balance is orchestrated by white adipose tissue (WAT) and brown adipose tissue (BAT)^2,3^. WAT is a specialized tissue for energy storage, in which triglycerides accumulate in large unilocular lipid droplets within adipocytes. Once needed, the white adipocytes can mobilize energy by hydrolyzing the triglycerides with the subsequent release of free fatty acids (FFAs), which are the key substrates for energy production in vertebrates during fasting^4^. In addition to its function in energy storage, WAT depots can also turn into energy dissipating tissues under cold exposure, adrenergic stimulation, and exercise, among others conditions^5^. This occurs mainly due to the recruitment of brown-like or beige adipocytes^2,6^. Browning involves the expression of the transcriptional regulator PR domain containing 16 (PRDM16), which is involved in the expression of thermogenic molecules, such as the PPAR-γ coactivator (PGC1α) and uncoupling protein-1 (UCP-1)^7^.

BAT is predominantly found in the interscapular region of rodents, although it can also be present in perivascular regions^8^. In adult humans, BAT is located in supraclavicular regions or around the neck^9^, and its discovery in humans emphasized the therapeutic potential of compounds that can alter energy expenditure and fuel metabolism in mammals, without the need for increasing physical activity, to address the global epidemics of obesity and diabetes^10,11^. Unlike white adipocytes, brown adipocytes store energy primarily to provide an intracellular source of fuel for thermogenesis^9^.

UCP-1 expression in adipocytes is the hallmark of BAT and is involved in the key step in thermogenesis, because it uncouples the mitochondrial respiratory chain, consequently generating heat (*i*.*e*., thermogenesis)^10^. Some known inducers of UCP-1 are cold exposure, food intake and the release of catecholamines^5^. These conditions promote β-adrenergic receptor stimulation, increase the cAMP, activation of protein kinase A (PKA) and the consequent induction of PGC1α, which in turn induces PPARα and lipolysis, and ultimately induces the expression of UCP-1^11^. The importance of lipolysis is related to the finding that FFAs are the main fuels for thermogenesis, and they also function as activators of UCP-1^12–14^. Therefore, due to its high metabolic capacity, BAT is an attractive target for the treatment of obesity, diabetes, and other metabolic disorders^14^.

During the last years, our group has explored the potential therapeutic applications of a unique polypeptide isolated from the venom of the South American rattlesnake *Crotalus durissus terrificus*, known as crotamine^15,16^. Crotamine is a 42 amino acid residues long polypeptide with a high content of basic residues (approximately 25% of the residues are positively charged), with a well-defined tridimensional structure that distributes the positively charged side chains on the surface, thereby contributing to an amphipathic feature for this molecule^17,18^. As these characteristics are commonly found in cell penetrating peptides (CPPs)^19^, we also evaluated and demonstrated the cell penetrating property of crotamine^20,21^. Then, we described the ability of crotamine to penetrate eukaryotic cells by endocytosis, in addition to functioning as a delivery vector and transporting therapeutic genes, and potentially also carrying other biologically active therapeutic molecules, with no detriment to its exceptional selectivity for actively proliferating cells^20,22^. These properties are important for the pleiotropic biological activities of crotamine, which include antimicrobial/antifungal^22^ and cytotoxic activities^23^, with a unique preference for highly proliferative cells, such as tumor cells^22,24^.

Despite the large spectrum of biological activities described to date for crotamine, knowledge about this polypeptide activity on healthy animal physiology remains very limited. Evidence that this toxin can act on metabolic pathways was first suggested by the observed decrease of body weight gain and increases in glucose tolerance when crotamine was orally administered daily to mouse models bearing subcutaneous melanoma tumors^24^. These effects of crotamine on body weight gain could potentially represent a limitation for using crotamine to treat cancer, as cachectic patients frequently experience unintended weight loss and episodes of hypoglycemia. Importantly, another group has demonstrated the ability of crotamine to induce insulin secretion in cultured pancreatic beta cells^25^. In the present study, the main objective was to investigate the metabolic effects of crotamine in healthy wild-type mice after 21 days of oral treatment, which was demonstrated by our laboratory to be an effective protocol for crotamine as an antitumor treatment agent^26^. Here, we found that crotamine induces the differentiation of brown adipocytes and stimulates basal energy expenditure, thereby reducing adiposity and improving glucose homeostasis.

## Results

### Chronic treatment with crotamine by gavage reduces body weight gain

**Figure 1:**
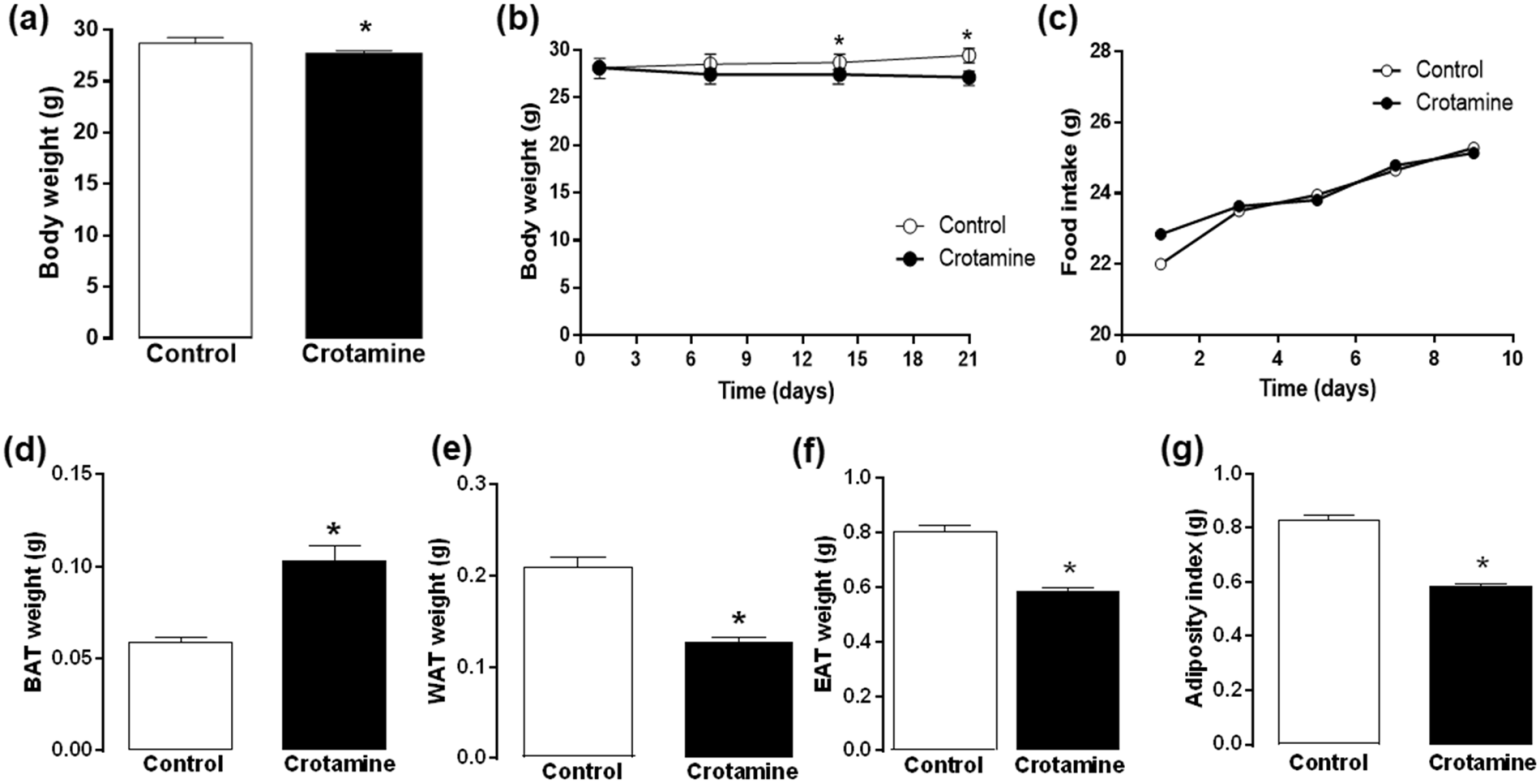
Body weight gain, food intake rate, fat depot weights and fat index of animals after oral treatment. (**a**) Mean body weight (**g**), (**b**) body weight gain (**g**), (**c**) food intake (**g**), (**d**) weight of brown adipose tissue (BAT, g), (**e**) weight of subcutaneous white adipose tissue (WAT, g), (**f**) weight of the epididymal WAT (EAT, (**g**), and (**g**) fat index. The results are presented as the mean ± SEM of three independent experiments (in which cohorts were composed of 5 animals in each group). *p < 0.05 for comparison between the treated and untreated control groups.

Mice treated for 21 days with crotamine (10 μg/animal/day) showed lower body weights compared to the control group that received only vehicle (Fig. 1a). Statistically significant differences in body weight gain of animals from crotamine-treated and control groups were first observed on the 14^th^ day of treatment, and this difference remained significant until the last day of treatment (Fig. 1b). Decreased body weight gain was not due to differences in eating behavior, as no significant differences in food intake was observed between the crotamine-treated and untreated control groups (Fig. 1c). After euthanasia, the fat depots were removed from the animals and weighed. A significant increase in BAT (Fig. 1d) and a significant reduction in subcutaneous WAT (Fig. 1e) and epididymal WAT depots were observed in crotamine-treated mice (Fig. 1f). In addition, the adiposity index (AI), which considers the sum of all isolated fat depots, was determined for both groups, showing significantly lower mean values for the group of animals treated daily with crotamine by oral gavage (Fig. 1g).

By contrast, this long-term treatment with crotamine did not significantly change the weights of liver, kidney, heart or brain (Table 1). Additionally, no evidence of animal body growth retardation was observed after 21 days of treatment with crotamine, as suggested by the absence of any significant differences in the weight of the skeletal muscles (gastrocnemius and soleus) or in the length of the bone (femur size) of the animals from the crotamine-treated or untreated groups (Table 1).

**Table 1:** Weight of the organs (liver, kidney, heart, and brain), skeletal muscle (gastrocnemius and soleus) and size (length) of the femur at the end of treatment with crotamine.

Tissue (g)	Control	Crotamine
Liver	1.381 ± 0.061	1.351 ± 0.052
Kidney	0.192 ± 0.002	0.172 ± 0.002
Heart	0.188 ± 0.013	0.175 ± 0.004
Brain	0.262 ± 0.025	0.306 ± 0.013
Gastrocnemius	0.300 ± 0.001	0.300 ± 0.001
Soleus	0.020 ± 0.001	0.016 ± 0.002
Femur length (cm)	1.61 ± 0.04	1.60 ± 0.04

Results are presented as the mean ± SEM of at least three independent experiments.

*p < 0.05 for comparisons between the treated and untreated control groups.

### Crotamine treatment improves glucose tolerance and increases insulin sensitivity

**Figure 2:**
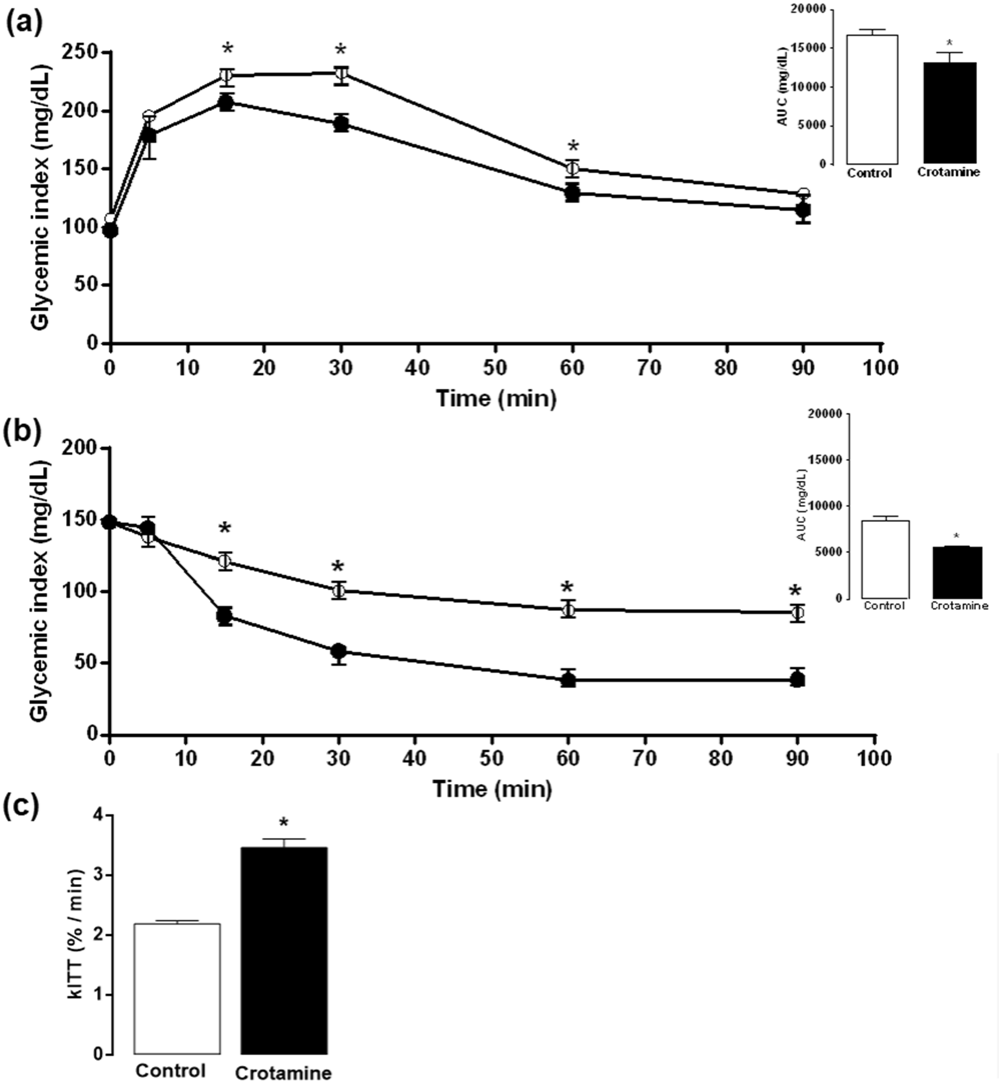
Evaluation of glucose tolerance and insulin sensitivity. (**a**) Glucose tolerance test (GTT) and (**b**) insulin tolerance test (ITT), at 0, 5, 15, 30, 60 and 90 min after stimulus. The respective area under the curve (AUC) values for each curve are presented in the insets. (**c**) The constant rate of glucose disappearance calculated from (**b**), the glucose decay index (kITT). The results are presented as the mean ± SEM of seven independent experiments. *p < 0.05 for comparison between the treated and untreated control groups.

A glucose tolerance test (GTT) demonstrated a clear improvement in glucose clearance after oral treatment with crotamine (Fig. 2a). The insulin tolerance test (ITT) showed increased insulin sensitivity in crotamine-treated animals (Fig. 2b).

Data obtained in the insulin tolerance test was also used to calculate the insulin-induced glucose decay rate (kITT), which is a *bona fide* marker of insulin action. Consistently, oral treatment with crotamine significantly increased the kITT (Fig. 2c).

### Reduced lipid levels in plasma and tissue damage markers in mice treated with crotamine

**Figure 3:**
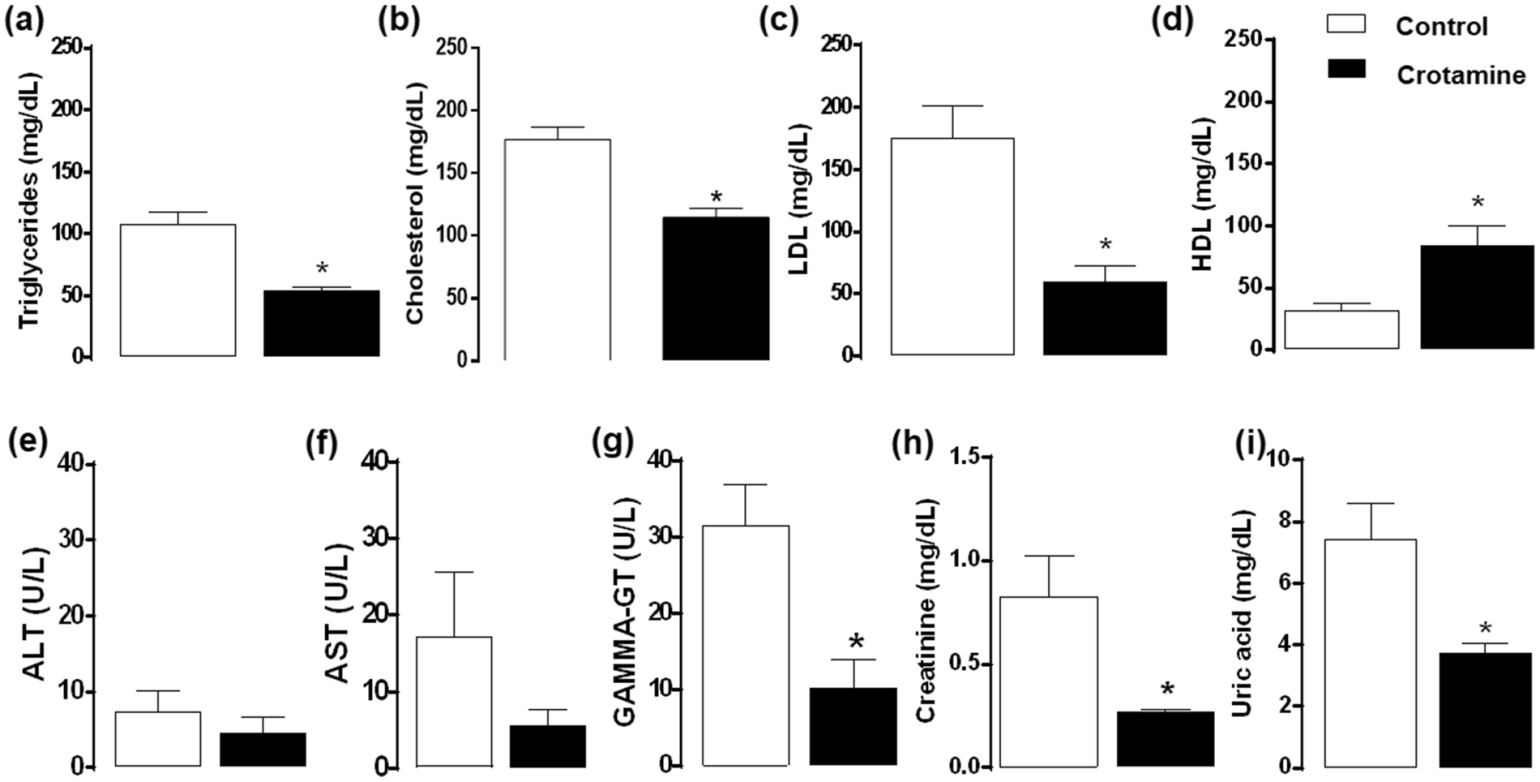
Biochemical evaluation of treated and untreated mice serum. (**a**) Triglycerides, (**b**) cholesterol, (**c**) LDL, (**d**) HDL, (**e**) ALT, (**f**) AST, (**g**) gamma-GT, (**h**) creatinine, and (**i**) uric acid. The results are presented as the mean ± SEM of six independent experiments. *p < 0.05 for comparison between the treated and untreated control groups.

Plasma was collected from mice soon after euthanasia, and parameters related to the metabolic status of the organism were measured. Significant reductions of triglycerides (Fig. 3a), total cholesterol (Fig. 3b), circulating low density lipoprotein (LDL) (Fig. 3c), and with only a trend towards increased high density lipoprotein (HDL) (Fig. 3d) levels were observed in crotamine-treated animals compared to the untreated control group receiving vehicle.

We also measured biomarkers of liver injury, and a trend towards a decrease of alanine aminotransferase (ALT) and aspartate aminotransferase (AST) levels was observed in the animals treated with crotamine (Figs 3e,f, respectively). Additionally, a significant decrease of gamma-glutamyl transpeptidase (gamma-GT) levels was observed in the plasma of crotamine-treated animals (Fig. 3g). Decreases in the levels of kidney injury biomarkers, *i*.*e*., creatinine (Fig. 3h) and uric acid (Fig. 3i), were also observed in the plasma of animals after 21 days of treatment with crotamine.

### Basal energy expenditure

To assess the possible determinants for the reduced weight gain, we performed measurements in a Comprehensive Lab Animal Monitoring System (CLAMS), which allowed the evaluation of both the oxygen uptake (VO_2_) and carbon dioxide production (VCO_2_) in a period of 24 h divided into two cycles, in which the ‘light cycle’ corresponds to the period of low activity and the ‘dark cycle’ corresponds to the period of high metabolic and physical activities of mice^27^.

**Figure 4:**
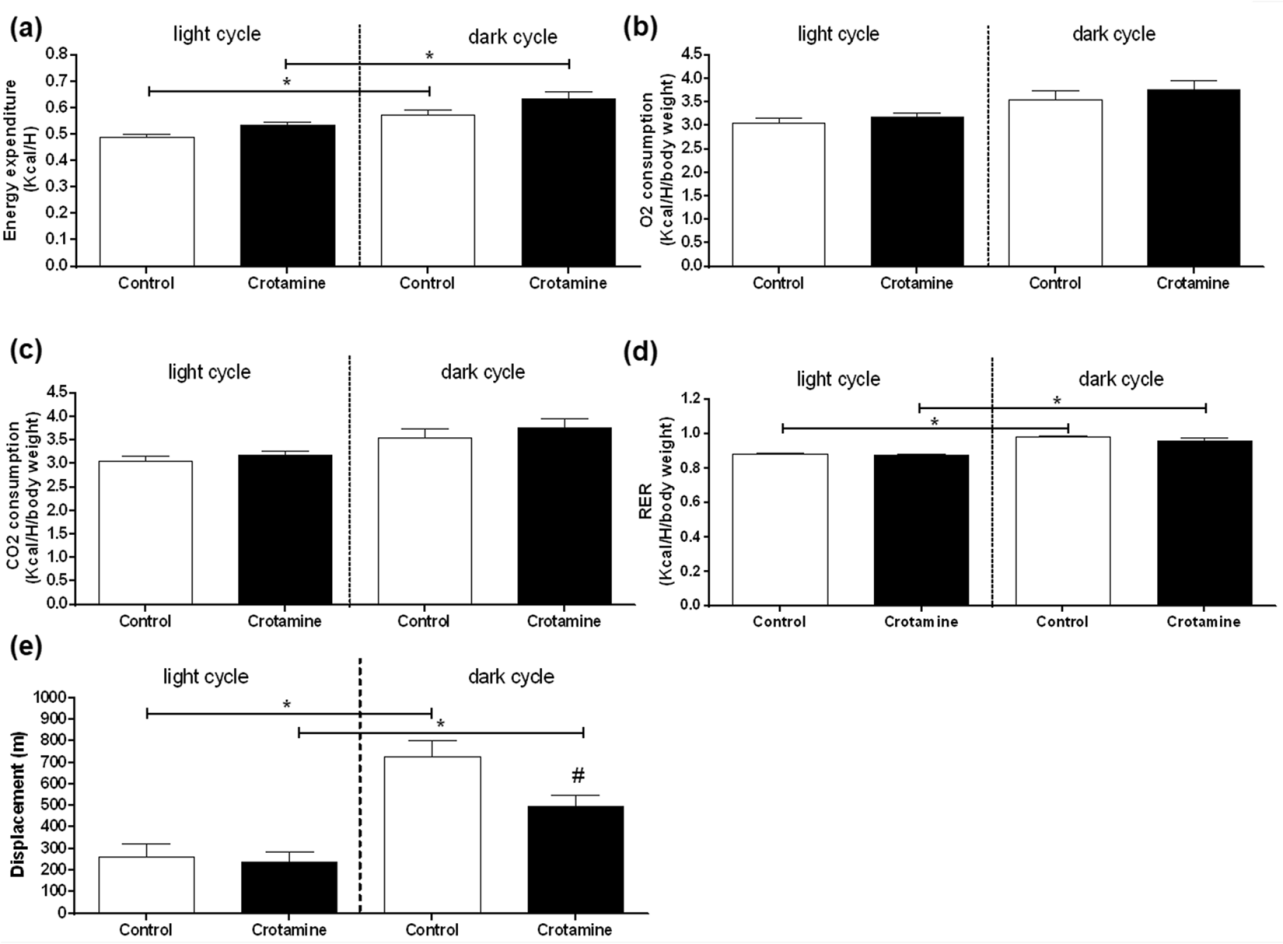
Basal metabolic profile assessed by calorimetry of treated and untreated animals. (**a**) Energy expenditure (HEAT). (**b**) oxygen consumption (VO_2_), (**c**) carbon dioxide consumption (VCO_2_), (**d**) respiratory exchange ratio (RER), and (**e**) ambulatorial displacement. The results are presented as the mean ± SEM of two independent experiments (in which cohorts were composed of 4 animals in each group). Statistical analysis was performed using one-way ANOVA test with Bonferroni post hoc analysis to compare the quantitative results among samples from treated and untreated groups at light and dark cycles (^#^*p* < 0.05). Student T-test (*p < 0.05) was also used to show significant differences between the light and dark cycles.

Increased energy expenditure denoted by increased heat production (HEAT) was noticed in the dark cycle compared to the light cycle, and the treatment with crotamine did not significantly affect this parameter (Fig. 4a). VO_2_ and VCO_2_ showed both a non-significant trend for higher values in the dark cycle compared to the light cycle, whereas no significant differences were observed for these parameters after treatment with crotamine (Fig. 4b,c).

Both groups consisting of animals treated with crotamine (experimental) or vehicle (negative control) presented respiratory exchange ratio (RER, which represents the substrate preference) values closer to 1 during the period of high metabolic activity (*i*.*e*., in the dark cycle) (Fig. 4d), which may suggest the preference for carbohydrates as an energy source. These values were significantly lower in the light cycle in both the experimental and negative control groups, and oral treatment with crotamine did not significantly influence RER values in either the dark or light cycles (Fig. 4d).

Ambulatory activity was also measured, and all animals (from both groups, *i*.*e*., crotamine-treated and untreated) showed higher locomotion in the dark cycle compared to the light cycle (Fig. 4e), as expected^27^. However, despite the increased energy expenditure, crotamine-treated animals showed a significant reduction in physical activity compared to the vehicle-treated control group (Fig. 4e). These data suggest that increased energy expenditure in crotamine-treated mice is likely due to an increase in the basal metabolic rate and/or heat production than altered physical activity.

### Effect *in vivo* of crotamine on the expression of thermogenic markers in BAT

**Figure 5:**
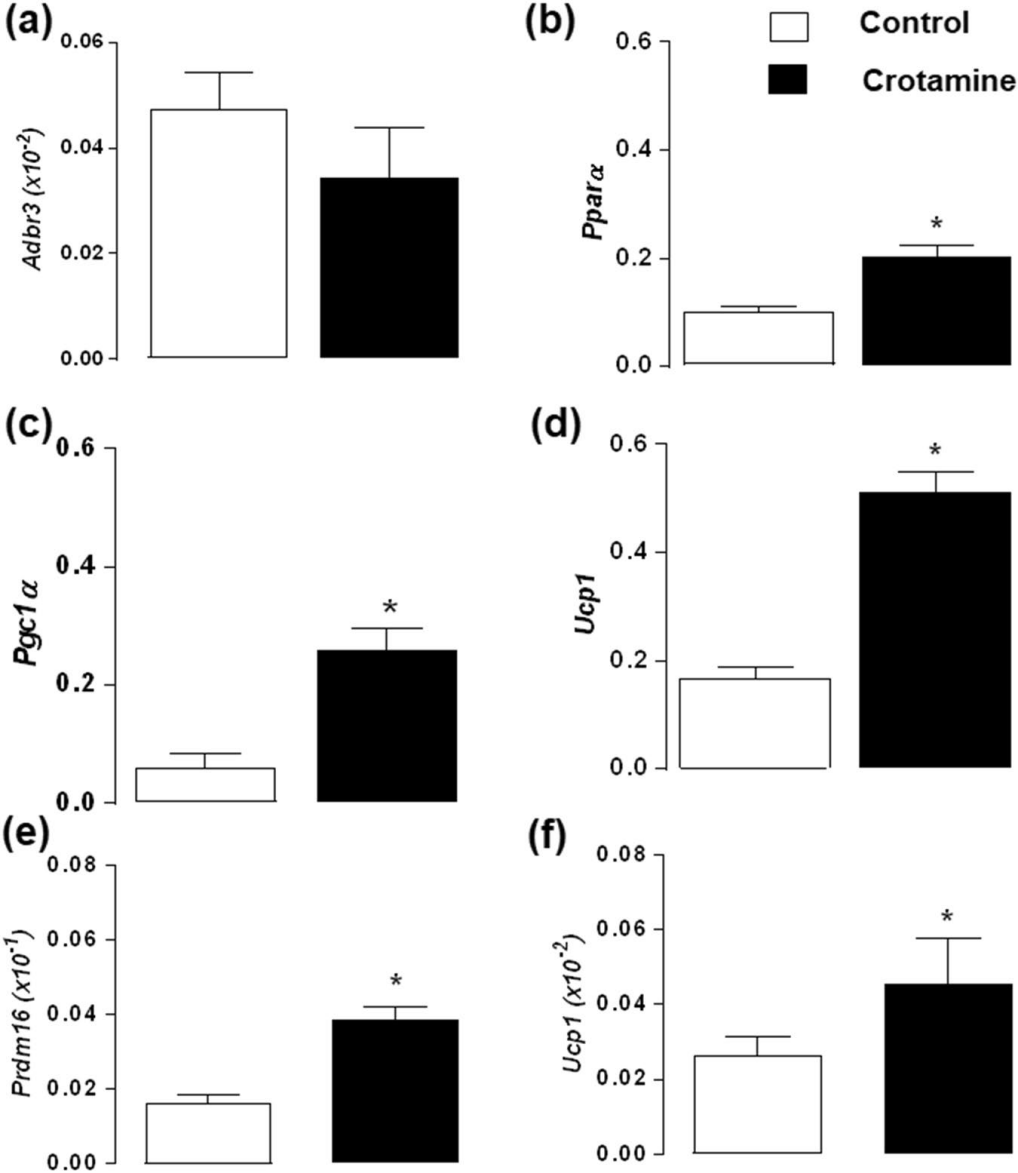
Evaluation of thermogenic marker expression in BAT and WAT of treated and untreated animals. Gene expression of (**a**) β-3 adrenergic receptor (*Adrb3*), (**b**) *Ppar*α, (**c**) *Pgc1α*, and (**d**) *Ucp1* in BAT, and gene expression of transcription factor (**e**) *Prdm16* and (**f**) *Ucp1* in WAT, all normalized against 18S. Data are the mean ± SEM of three independent experiments. *p < 0.05 for comparison between the treated and untreated control groups.

Thermogenic marker gene expression was analyzed and normalized to 18S using the total RNA extracted from the BAT of animals treated or not treated with crotamine. Treatment with crotamine by the oral route for 21 days showed a trend towards a reduced the expression of the beta-3 adrenergic receptor mRNA (*Adrb3*, Fig. 5a). By contrast, the expression of *Pparα* (Fig. 5b), *Pgc1α* (Fig. 5c) and *Ucp-1* (Fig. 5d), which is the primary target for inducing thermogenesis in BAT, were all significantly increased in mice after treatment with crotamine for 21 days.

### Crotamine effect on the *in vivo* recruitment of beige adipocytes in the subcutaneous WAT

To assess whether treatment with crotamine could induce beige adipocyte recruitment in WAT, two of the major markers of “browning”, *i*.*e*., *Prdm16* and *Ucp-1*^28–30^, were measured in subcutaneous WAT. Consistent with our hypothesis, a significant increase in the expression of beige adipocyte recruitment marker genes was observed in animals receiving crotamine by the oral route for 21 days (Fig. 5e–f).

### *In vitro* differentiation and maturation of cultured brown preadipocytes in the presence of crotamine

**Figure 6:**
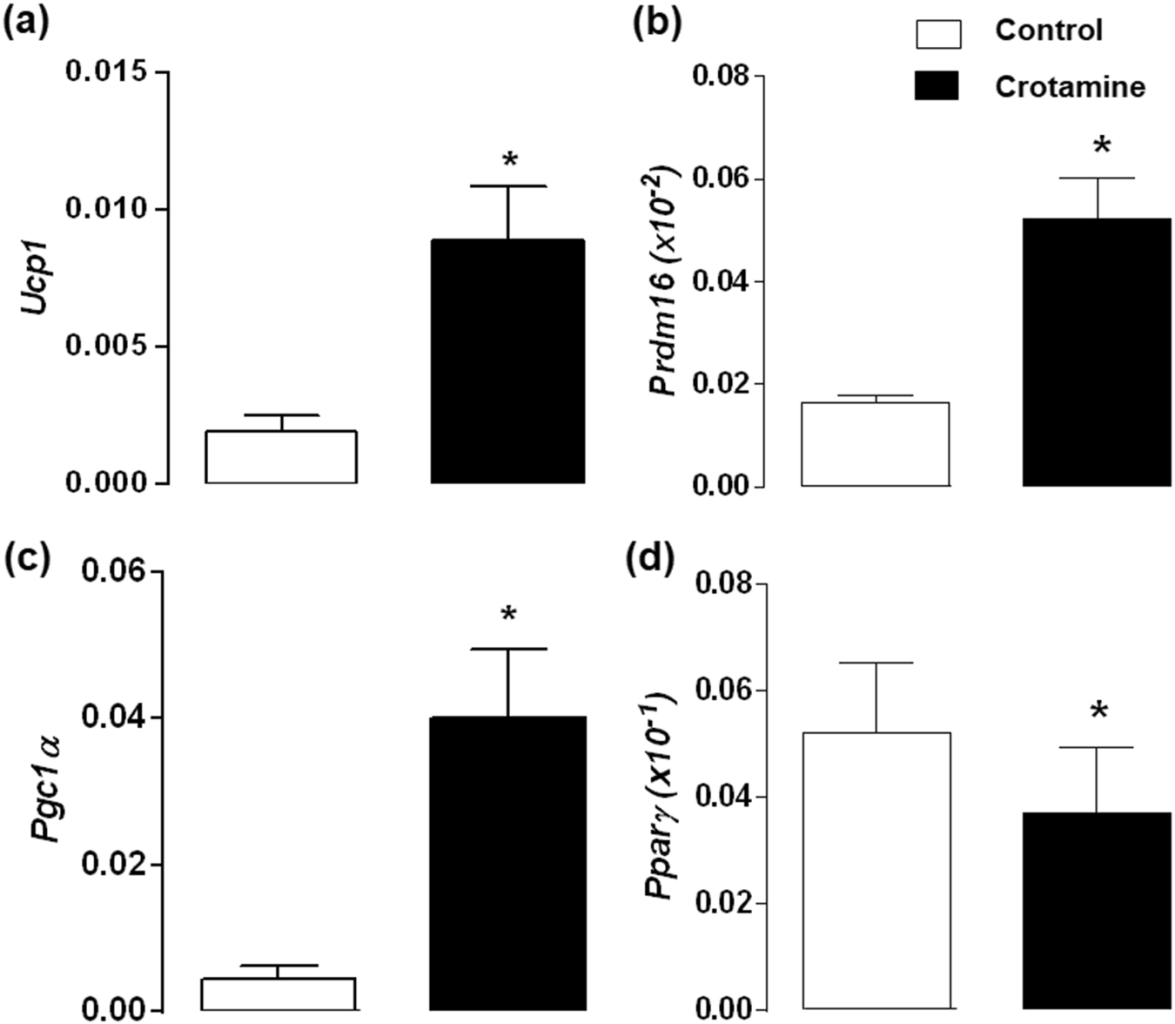
Evaluation of expression of markers for differentiation and activation of brown adipocytes (9B). Gene expression of the transcription factors (**a**) *Prdm16* and (**b**) *Pparγ*, (**c**) *Pgc1*α, and (**d**) *Ucp1* normalized against 18S is shown, and the results are presented as the mean ± SEM of five independent experiments. *p < 0.05 for comparison between the treated and untreated control groups.

**Figure 7:**
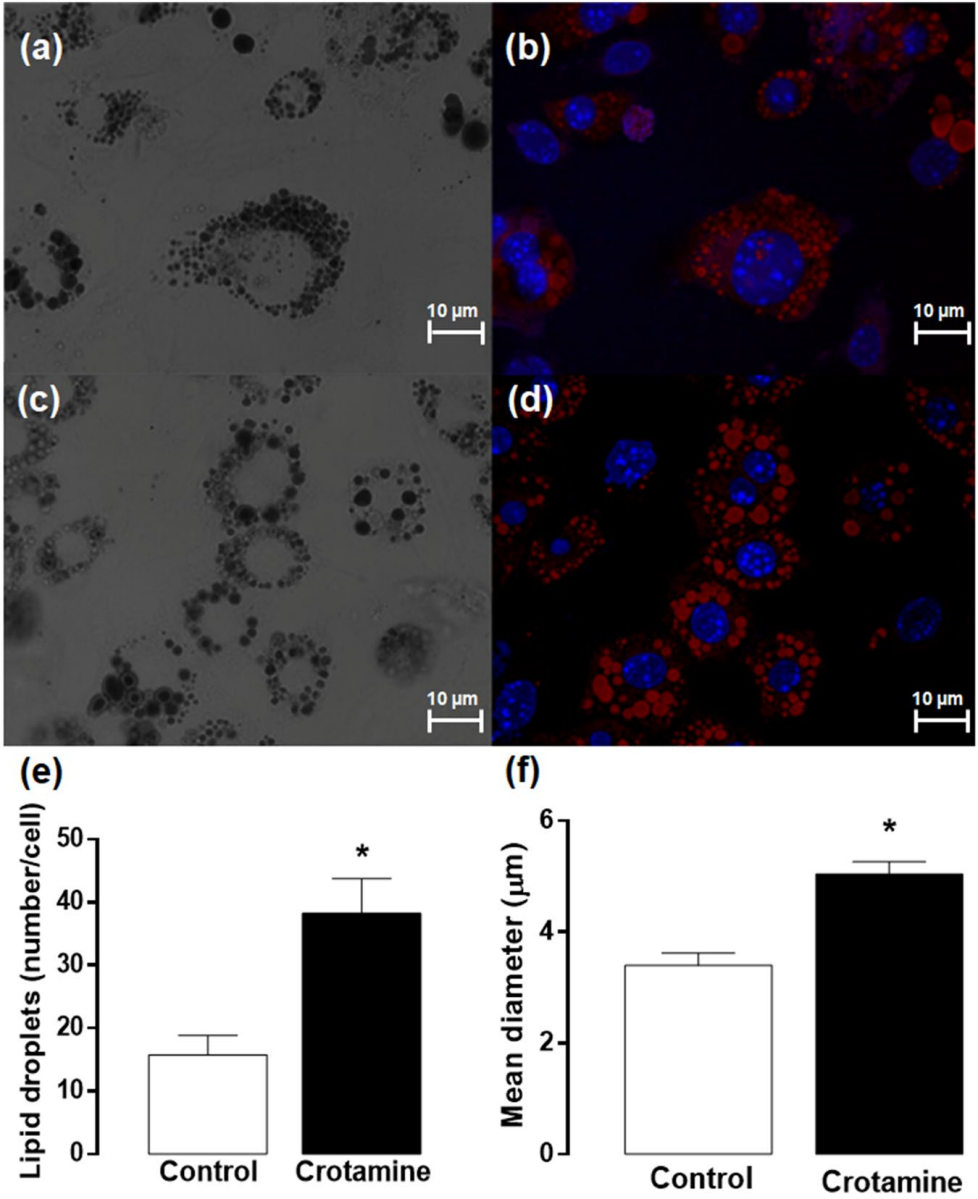
Confocal imaging of differentiated adipocyte cells. Immortalized brown preadipocytes were induced to adipocyte differentiation in the absence (**a** and **b**) or presence (**c** and **d**) of crotamine (5 μM). Differential interference contrast (DIC) imaging (**a** and **c**) allows the morphological visualization of cells and of lipid vesicles, whereas the nucleus of the cells and lipid vesicles are shown by DAPI (λ_Ex_ 405 nm/λ_Em_ 420–470 nm) and red O oil (λ_Ex_ 543 nm/λ_Em_ 555–625 nm) staining, respectively (b and d). Scale bars = 10 μm. The average number of lipid droplets per cell (**e**) and the mean value of diameter of lipid droplets (**f**) were determined by using ImageJ software (N = 50 cells). *p < 0.05 for comparison between the treated and untreated control groups.

The cell autonomous effects of crotamine on brown adipocytes was assessed using immortalized 9B brown preadipocyte cultured cells, which were induced to differentiation in the presence of crotamine (5 μM), which was added at the first day of the differentiation process that lasted for 8 days. This resulted in a significant increase in the expression of *Ucp-1* (Fig. 6a), *Prdm16* (Fig. 6b) and *Pgc1α* (Fig. 6c). By contrast, a significant decrease in the expression of a pan-adipocyte marker *Pparg* was observed upon treatment with crotamine (Fig. 6d), suggesting that the effect of crotamine was not due to a general increase in the differentiation process. In addition, confocal imaging analysis of these immortalized brown preadipocyte cells (Fig. 7a–d) did show a significant increase in the number of lipid droplets per cell and mean size/diameter of the lipid droplets (Fig. 7e–f), when adipocyte cells were induced to differentiation in the presence of crotamine (5 μM).

## Discussion

Natural products have significantly contributed to the discovery of several new compounds with potential as therapeutics or as structural leads for the development of new drugs^31^. Some natural compounds, such as polyphenols and flavonoids, can reduce the absorption of lipids^32^ to induce lipolysis and subsequently decrease adiposity^33^. For example, green tea and caffeine were described to induce energy expenditure by increasing *Ucp*-1 expression in BAT^34^. However, natural compounds isolated from venomous animals have been less studied in this field, and an unexplored collection of molecules with potential to counteract obesity and ameliorate its complications remains unexplored. Previous observations that crotamine inhibits weight gain in animal models of melanoma^24,26^ led us to explore the potential effects of crotamine on metabolism.

Here, we demonstrate that crotamine stimulates brown/beige adipocyte differentiation/activation both *in vivo* and *in vitro*. In B16F10 and CHO-K1 cells, crotamine was shown to promote Ca^2+^ release from intracellular stores^21,35^. Interestingly, increases in intracellular free Ca^2+^ activates calcineurin and inhibits ERK phosphorylation, which in turn inhibits brown adipocyte differentiation^36,37^. By contrast, we show that crotamine promotes activation of BAT function and differentiation, suggesting that this polypeptide does not act through calcineurin and ERK. However, more experiments are required to test the mechanism by which crotamine acts directly in preadipocytes to promote brown adipocyte differentiation. Moreover, the mechanisms of action of crotamine in eukaryotic cells at sub-lethal concentrations remain largely unexplored.

*In vivo*, the direct effect of crotamine on browning is likely potentiated by other systemic, indirect actions. In the present work, expression of the β3 adrenergic receptor mRNA was decreased in BAT upon the treatment of mice with crotamine for 21 days by the oral route. We hypothesize that the adrenergic tonus of crotamine-treated mice could be increased, sustaining thermogenic activity and promoting BAT expansion, in which a given receptor expression is decreased in response to the high levels of its ligand^38–40^.

Interestingly, crotamine may also play a role in glucose tolerance by affecting insulin release, as crotamine affects K^+^ channels and intracellular Ca^2+^ pool dynamics^21,41,42^ and controls the secretion of insulin-containing intracellular vesicles^25,43^.

In this study, the ability of the polypeptide crotamine to act in BAT and WAT of mice to inhibit body weight gain and to stimulate a pro-thermogenic profile that favors energy expenditure was shown. Therefore, we propose a direct effect of crotamine in brown preadipocytes, in addition to possible systemic effects *in vivo*. By inducing browning, crotamine leads to reduced body weight gain, which in turn favors insulin sensitivity. Brown/beige adipocytes also consume high amounts of glucose and lipids, which explains the improved glucose tolerance and reduced triglyceride levels, respectively, in animals treated with crotamine. BAT plays a fundamental role in maintaining body temperature by producing heat, and crotamine effects on the metabolism of wild type healthy mice (without tumor) inducing browning as shown in the present study precludes any possible cachexia-like effect of this polypeptide.

Cancer cachexia is a paraneoplastic syndrome characterized by body weight loss, muscle wasting, adipose tissue atrophy and inflammation^44^, in which muscle wasting has emerged as a principal component of cancer cachexia, leading to progressive impairment of work capacity^45^. Here, we showed that despite the significant decrease of body weight gain after oral treatment with crotamine, no significant body weight loss or decrease in skeletal muscle, brain, kidney or heart weight was observed. In addition, the size of the femur bone of animals was also the same regardless of treatment with crotamine. In addition to the loss of body weight, most advanced cancer patients frequently suffer loss of appetite (anorexia), which contributes to cachexia, whereas mice treated with crotamine only have decreased body weight gain but with no significant change in food intake. Additionally, analysis of biochemical biomarkers for liver and kidney dysfunction showed a trend towards decreased levels after treatment with crotamine, suggesting no potential damage to liver or kidney functions. Interestingly, antioxidants that protect and improve mitochondrial respiration reduced these biochemical biomarkers of organ dysfunction, including ALT and creatinine^46^, and crotamine action on mitochondria was also previously suggested by us^21^.

Taken together, our data open new roads for the exploration of novel pharmacological leads based on crotamine structure to treat obesity or dysfunctions associated with weight gain and insulin resistance. However, further studies are necessary to demonstrate the efficacy of crotamine in animal models of diseases, such as obesity or diabetes. Similarly, the mechanism(s) underlying these effects must be better elucidated by us at the molecular level, with the main focus on the direct action of crotamine direct on preadipocytes to identify possible molecular targets or pathways with therapeutic value.

## Materials and Methods

### Purification of crotamine

The venom of *Crotalus durissus terrificus* was extracted from rattlesnakes kept captive at the Ribeirão Preto Medical School (FMRP) Serpentarium of University of São Paulo (USP), and native crotamine was purified according to the previously described protocol^47^.

### Animals

Male C57BL/6 mice, 12 weeks old, obtained from the Animal Experimentation Laboratory of INFAR at the Federal University of São Paulo (UNIFESP/EPM - SP, Brazil) were divided in 2 experimental groups: the control sham group, treated with vehicle (n = 8), and the experimental group, treated with crotamine (n = 8). The animals were maintained under controlled temperature (19 °C), light/dark cycle every 12 h, and they were supplied with water and food *ad libitum*. All procedures with the animals were in accordance with the standards described in the Guidelines for Ethical Conduct in the Care and use of Animals, the American Psychological Association* and the Guideline of the Committee on Care and Use of Laboratory Animal Resources of the National Research Council of the United States of America. The Research Ethics Committee of UNIFESP approved all procedures performed in this study (CEUA No. 1361030315).

### Treatment of animals

The animals from the treated group received 10 µg of crotamine in 100 µL of filtered water daily through an orogastric tube (gavage). The control group also received 100 μL of filtered water by oral administration. The treatment lasted for 21 days, as adopted previously for antitumor treatment^24^.

### Body weight gain and food intake evaluation

During the experimental period, the animals were weighed weekly using a semi-analytical balance. The dietary intake was calculated weekly as the difference of the initial quantity supplied and the remaining amount of food (in grams).

### Insulin Tolerance Test (ITT)

First, a sample of blood (1 drop of approximately 10 µL) was collected from the animals fasted for 8 h after making a small cut (of approximately 3 mm) at the extremity of the animal tail, at time zero of the experimental procedure. After the first collection (time zero), regular insulin (0.5 U/kg, Biobrás, São Paulo, Brazil) was injected, and blood samples were collected after 5, 10, 15, 30, 60 and 90 min for glucose measurements using the Accu-check blood glucose meter (Roche Diagnostics, Mannheim, Germany).

### Glucose tolerance test (GTT)

Two days after the ITT test, a sample of blood (1 drop approximately 10 µL) was collected from each animal fasted for 8 h through a new cut of approximately 3 mm at the extremity of the animal tail, to determine the glucose concentration in the blood at time 0 (or namely, ‘background level’ for the fasted animal). Then, a glucose solution was administered by intraperitoneal (IP) injection in a dose of 1 g of glucose/kg of animal body weight. Blood samples from each animal were collected again from the same tail cut at 5, 10, 15, 30, 60 and 90 min after glucose injection for the measurement of blood glucose levels using an Accu-check blood glucose meter system (Roche Diagnostics). The obtained values were also used for construction of the tolerance curve.

### Biochemical analysis of the plasma

The total volume of blood collected from each animal (~ 0.5 mL) was placed in single microtube containing EDTA that was centrifuged for 10 min at 300 × g at room temperature for plasma fraction (supernatant) collection. Aliquots of plasma were stored in 0.2 mL microtubes at −80 °C until analysis. Specific kits, all from BIOCLIN (São Paulo, Brazil) were used to determine the plasma concentration of triglycerides (Triglicerides Monoreagente – K117), total cholesterol (Colesterol Monoreagente – K083), uric acid (Ácido Úrico Monoreagente – K139), creatine (Creatinina Enzimática – K161), alanine aminotransferase (ALT, Transaminase ALT (TGP) Cinética – K049), aspartate aminotransferase (AST, Transaminase AST (TGO) Cinética – K048) and gamma-glutamyl transpeptidase (gamma-GT, Gama GT Cinético – K080), which were used according to the manufacturer’s instructions.

### Basal metabolic rate

The basal metabolic rate of each animal was assessed 14 days after experimentation using an indirect open circuit calorimeter Oximax model (Columbus Instruments, Ohio, USA). First, the animals were acclimated for 24 h in cages (one animal/cage) under 12 h light/dark cycle with food and water *ad libitum*, and they were kept under these conditions for the experimental period, which lasted for over 24 h. The animals were subjected to non-invasive monitoring of gas exchange and physical activity. The rates of oxygen consumption (VO_2_) and CO_2_ production (VCO_2_), as well as respiratory exchange ratio (RER), were determined following the manufacturer’s instructions. Animal displacement was measured using light sensors installed at the base of the cages.

### Tissue collection and adiposity index evaluation

Immediately after euthanasia, a midline laparotomy was performed in each animal for the extraction of white adipose tissues (epididymal, perirenal and subcutaneous), brown adipose tissue (BAT), kidney, heart, brain, liver, and gastrocnemius and soleo skeletal muscles. The tissues and organs were individually weighed using an analytical balance. The sum of the fat tissue weight was normalized by the average body weight of the respective group to determine the adiposity index (AI), using the Equation (1) below.

1 $${\rm{AI}}={{\rm{WAT}}}_{{\rm{sub}}}+{{\rm{WAT}}}_{{\rm{epi}}}+{\rm{BAT}}/{\rm{mean}}\,{\rm{body}}\,{\rm{weight}}$$

### Brown preadipocyte cell culture and confocal microscopy

**Figure 8:**
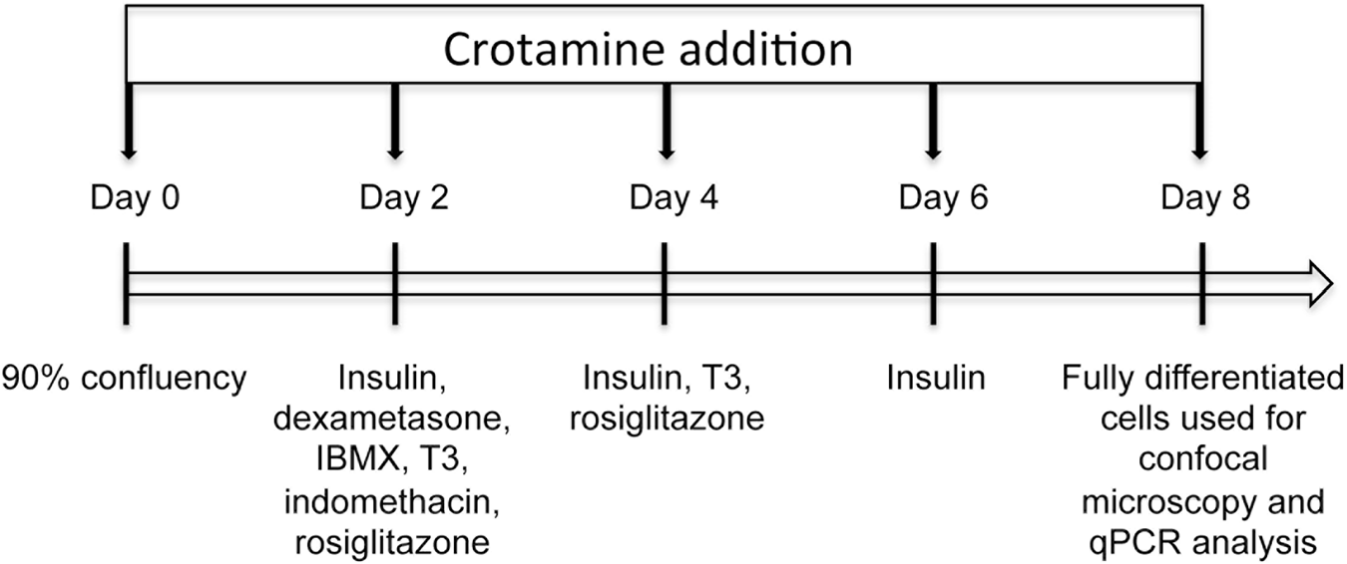
Schematic representation of the cell differentiation process. Immortalized brown preadipocytes cell line 9B were induced to adipocyte differentiation in the absence or presence of crotamine (5 μM). Crotamine was added in each day of medium change for replacement of the different drugs of the cocktail for differentiation, as indicated by the vertical arrows.

The brown preadipocyte cell line 9B are immortalized dicer^fl/fl^ brown preadipocytes^48^. These cells were isolated from the intrascapular brown adipose tissue of newborn dicer^fl/fl^ mice upon collagenase digestion (1.5 mg/mL; Worthington Biochemical, Lakewood, NJ, USA). After two days in culture, the cells were immortalized using retrovirus harboring the pBabe SV40 Large T antigen puromycin vector. Dicer^fl/fl^ mice behave similar to wild type mice as do the cells, which express a high levels of UCP1 and are highly uncoupled (unpublished data). These cells were grown and expanded with DMEM medium (Sigma Aldrich, St. Louis, USA) and supplemented with 10% fetal bovine serum (FBS). Then, the cells were cultured at 37 °C with 5% CO_2_, and treated with a cocktail of drugs for differentiation (insulin, dexamethasone, rosiglitazone, IBMX (3-isobutyl-1-methylxanthine), indomethacin, T3 and isoproterenol) for 8 days in the presence of crotamine (5 μM) that was replaced every 2 days with cells washing and medium changes (as schematically demonstrated in Fig. 8).

After the differentiation process, the cells were collected and stored at −80 °C until use or they were cultured on cover glasses mounted with anti-fading medium containing DAPI for imaging analysis by confocal microscopy in a Leica TCS SP8 confocal microscope (Leica Microsystem, Wetzlar, Germany) using an oil immersion objective of 63× (as detailed in the figure legend), at wavelengths λ_Ex_ 405 nm/λ_Em_ 420–470 nm for DAPI fluorescence. Lipid accumulation was visualized at day 8 of differentiation by red O oil staining (Aldrich Sigma). Cells were washed once with PBS and were fixed by immersion in PBS + 10% formaldehyde for 15 min, at 4 °C. Cells were then stained with filtered red O oil solution (5 g/L in isopropyl alcohol) diluted 2-fold in water. After incubation for 1 h at room temperature, the cells were rinsed several times with distilled water to remove the excess stain and precipitates. For imaging analysis by confocal microscopy on a Leica TCS SP8 confocal microscope (Leica Microsystem), red O oil were detected at wavelengths of λ_Ex_ 543 nm/λ_Em_ 555–625 nm. For the measurement of the apparent lipid droplet diameter and determination of number of lipid droplets per cell, micrographs obtained by confocal microscopy were analyzed using ImageJ software^49^.

### Quantitative RT-PCR

The mRNA was extracted from the BAT and WAT samples using Trizol® reagent. Then, the cDNA was synthesized using the SuperScript First-Strand Synthesis System for RT-PCR (Invitrogen by Life Technologies, Carlsbad, California, USA). The gene expression rate was then evaluated by quantitative real-time PCR (qPCR), and each reaction mixture containing 250 nM of each primer (sense and antisense), 25 ng of cDNA and 1 × Maxima SYBR-Green Master Mix in a final volume of 10 μL was analyzed in a Stratagene Mx 3000 P qPCR system (Thermo Fisher Scientific, Waltham, MA, USA) under the following amplification conditions: 50 °C-2 min, 95 °C-10 min, 40 cycles of 95 °C-15 s, 60 °C-20 s and 72 °C-30 s. Dissociation protocols were used to evaluate the efficacy of the primers for specifically amplifying the target genes. The sequence of the primers for the genes of interest and a housekeeping gene (18S) as the endogenous control used in our experiments are shown in Table 2. The threshold cycle (*C*_*T*_) values derived from the real-time PCR assay for each gene were divided by the *C*_*T*_ values of the internal control 18S, and these ratio values were used to prepare the graphics.

**Table 2:** Sequences of the oligonucleotides.

Gene	Forward	Reverse
36B4	GCAGACAACGTGGGCTCCAAGCAGAT	GGTCCTCCTTGGTGAACACGAAGCCC
18 S	CTCAACACGGGAAACCTCAC	CGCTCCACCAACTAAGAACG
Adrenergic Recpt β3	CCTTCCGTCGTCTTCTGTGT	CCTGCAAAAACGGAACAAT
PPARα	TCAGCTCTGTGGACCTCTCC	ACCCTTGCATCCTTCACAAG
PPARγ	GCCCTTTGGTGACTTTATGGA	GCAGCAGGTTGTCTTGGATG
PGC1α	CCCTGCCATTGTTAAGAC	TGCTGCTGTTCCTGTTTTC
UCP-1	AGCAGACATCATCACCTTCC	TTCGGCAATCCTTCTGTCTT
PRDM16	ACATCCGTCTAGCGTGTTCC	GCACCAACAGTTCCTCTCCA

Oligonucleotides used for the analysis of expression in brown adipose tissue (BAT) and subcutaneous adipose tissue of animals treated with crotamine by the oral route for 21 days.

### Analysis of data

The results presented here are expressed as the mean ± standard error of the mean (SEM). Statistical analysis was performed using two-way ANOVA test with Bonferroni post hoc analysis to compare the quantitative results among multiple samples. Statistical differences between two means were determined by the Student “t” test. The significance level to reject the null hypothesis was set at 5% (*p* < 0.05).

### Data availability statement

The datasets generated during and/or analyzed during the current study are available from the corresponding author upon reasonable request.
